# Identification of more objective biomarkers for Blood-Stasis syndrome diagnosis

**DOI:** 10.1186/s12906-016-1349-9

**Published:** 2016-09-22

**Authors:** Jiangquan Liao, Yongmei Liu, Jie Wang

**Affiliations:** 1 Guang’anmen Hospital, China Academy of Chinese Medical Sciences, No.5 Beixiange Street, Xicheng District, Beijing, 100053 China; 2 Graduate School, Beijing University of Chinese Medicine, No.11 Beisanhuan East Road, Chaoyang District, Beijing, 100029 China

## Abstract

**Background:**

Blood-stasis syndrome (BSS) is one of the Traditional Chinese medicine (TCM) syndrome differentiations that are commonly seen in stroke and ischemic heart diseases; however, the BSS differentiation criterion is not standardized. More objective biomarkers for BSS diagnosis are needed.

**Methods:**

Acute ischemic stroke (AIS) or unstable angina (UA) patients with BSS and healthy controls were enrolled. The miRNA and mRNA expression profiles of UA patients and AIS patients were compared to those of healthy controls to identify the differentially expressed miRNA and mRNA of BSS. Bioinformatics analysis was used to identify significantly deregulated miRNAs and mRNAs correlated to BSS. QRT-PCR was performed to validate the bioinformatics analysis results.

**Results:**

Approximately 401 mRNAs and 11 miRNAs were differentially expressed in both UA and AIS patients compared to healthy controls. Gene ontology (GO) functional analysis was performed, and multiple GO terms were enriched. Among the overlapping DE miRNAs and mRNAs, miR-146b-5p, -199a-5p and 23 targeted mRNAs were pivotal genes in the BSS genomic characteristics. These 2 miRNAs and 23 mRNAs formed network-type biomarkers for BSS.

**Conclusions:**

The genomic characteristics of BSS were shown in this study. miR-146b-5p, -199a-5p and the 23 targeted mRNAs formed a diagnostic network for BSS. Further improvement and validation of this diagnostic network might lead to more objective diagnostic criteria for BSS.

## Background

Atherosclerosis (AS) is a pathological process that could affect the systemic blood vessels. Cerebrovascular- and cardiovascular-related AS could lead to severe diseases, such as stroke and myocardial infarction. Stroke and ischemic heart disease (IHD) are the major causes of death and long-term disability worldwide. According to World Health Organization (WHO) [1], IHD and cerebrovascular disease are the top 2 leading causes of death at all ages, contributing to 12.2 and 9.7% of all deaths. Despite these concerns and efforts devoted to preventing and treating cardiovascular and cerebrovascular diseases, the death rate increased significantly from 1990 to 2013 [2]. Continuous research on the underlying mechanism and treatment optimization are needed for AS-related stroke and IHD.

The pathology and mechanism of stroke and IHD are similar to a certain extent. Ischemic stroke (IS) and IHD are mainly caused by local vascular AS. Local inflammation, increased blood viscosity, hemodynamic abnormalities and hyperlipidemia could accelerate the stimulation of lipids and deteriorate the vascular function. Even with a comprehensive understanding of AS, the prevention and treatment of IS and IHD are not currently possible. Traditional Chinese Medicine (TCM) might help to combat stroke and IHD. It has been proven that TCM could improve the clinical symptoms and prognosis of IS and IHD [3–8]. The utilization of TCM in the world is increasing as the efficacy of TCM has been acknowledged [9, 10].

To maximize the efficacy of TCM treatment, an accurate estimation of syndrome differentiation is crucial because it guarantees the accurate judgment of the disease and the application of the herbal formula or patent medicine. Syndrome differentiation is also a large challenge for practitioners to fully apprehend TCM because the standard of syndrome differentiation is either not determined or too subjective. According to a number of surveys, the main syndrome of IS and IHD is Blood-Stasis syndrome (BSS). One web-based survey in Korea reported that the most common difficulty (52.5 %) in diagnosing BSS for doctors in Korea was “the lack of an objective measurement method,” and more than half (88.9 %) of the participants thought that an objective diagnostic method for BSS was necessary [11]. Current estimations of BSS are mostly based on clinical symptoms [11, 12], and seeking a reliable biomarker for BSS and other syndromes is one of the hot spots in TCM research [13–15].

Messenger RNA (mRNA) and microRNA (miRNA) might be potential biomarkers for BSS. miRNA is an endogenous, non-protein coding, single-stranded, small RNA that is generally regarded as negative regulator of gene expression by inhibiting translation and/or promoting mRNA degradation [16]. Research has shown that the expression of mRNA and miRNA varies in different syndromes of diseases [17–22], and the patterns and interactions between mRNA and miRNA may provide an approach to estimate the syndrome differentiation.

In this study, we used microarray profiling and bioinformatics to investigate miRNA-mRNA expression patterns in BSS patients to gain insight into BSS and provide potential biomarkers for BSS in the treatment of IS and IHD such that the utilization of TCM would be relatively convenient and more accurate. The miRNA and mRNA expression profiling of BSS unstable angina (UA) patients and acute ischemic stroke (AIS) patients were compared to healthy controls to identify the differentially expressed miRNA and mRNA of BSS. Bioinformatics analysis was used to identify significantly deregulated miRNAs and mRNAs involved in the pathogenesis of BSS.

## Methods

### Participants and recruitment

Twenty patients with UA and 20 patients with AIS were recruited in Guang’anmen Hospital, Beijing, China. All of their TCM syndrome differentiations were BSS. The diagnosis of coronary artery disease (CAD) was confirmed in all UA patients by coronary angiography, with at least one vessel lesion (>50 % narrowing of luminal diameter). All UA patients met the American College of Cardiology/American Heart Association (ACC/AHA) criteria for UA [23] and experienced ischemic chest pain within 48 h before recruitment, including angina pectoris with an accelerating pattern or prolonged duration (>20 min) or recurrent episodes at rest or with minimal effort, but with no evidence of enzymatic criteria. ECG showed ST segment deviation and/or T wave inversion. For patients undergoing percutaneous coronary intervention, blood samples were taken before this procedure.

All AIS patients met the American Heart Association/American Stroke Association (AHA/ASA) criteria for AIS [24], as confirmed by brain CT or MRI scan. All AIS patients had experienced focal neurological symptoms within 48 h before recruitment, including dysphagia, dysarthria, hemianopsia, limb weakness, ataxia, sensory loss and spatial neglect, but their consciousness was generally clear or slightly damaged.

The diagnosis of BSS was based on a grading system drafted by the committees of the International Conference on Blood Stasis Syndrome (October, 1988, Beijing, China), which has been used in China for more than 2 decades [12, 25]. It has 33 items for diagnosis, including symptoms, signs, and laboratory tests (Table 1). If the grading point of BSS in a patient is greater than 19, the patient should be diagnosed with BSS. The diagnosis of BSS was made by 3 appointed senior TCM practitioners. Patients were included in the study only if the 3 practitioners reported consistent results, which ensured that all of the selected patients had the typical manifestations of BSS.

**Table 1:** The grading system in quantifying blood stasis syndrome diagnosis standards

Signs and symptom	Point
Purple tongue	(less severe) 8, (more severe) 10
Resistance to pressure in lower abdomen	(less severe) 8, (more severe) 10
Choppy pulse	10
Dark stool (Melena)	10
Pathogenic nodules	10
Distended veins under tongue	(less severe) 8, (more severe) 10
Irregular pulse	8
No pulse	10
Distended veins in abdominal wall	10
Hypodermal ecchymoses	(less severe) 8, (more severe) 10
Dark menstrual blood with clots	(less severe) 8, (more severe) 10
Persistent angina pectoris	10
General fixed pain	8
Dark red lips and gums	6
Small vessels	5
Numb extremities	5
Surgery history	5
Mucosal membrane of palate (+)	(less severe) 4, (more severe) 5
Paralysis in extremities	(less severe) 5, (more severe) 7
Psychiatric abnormality	(Irritability) 4, (Mania) 8
Rough skin	(less severe) 4, (more severe) 5
Complete blood viscosity (+)	10
Blood plasma viscosity (+)	5
External clot net weight (+)	10
External clot total weight (+)	8
Increase in platelet aggregation	10
Abnormality in blood clot elasticity	8
Microcirculation obstruction	10
Hemodynamics obstruction	10
Decrease in fiber dissolution activity	10
Resistance in platelet release	10
Pathogenic scan (+) for blood stasis	10
Blood vessel obstruction by new technology analysis	10

Grades >19 points are categorized as Blood-Stasis syndrome

Patients who had received thrombolytic therapy in the previous month or with MI, heart failure, valvular heart disease, dilated cardiomyopathy, malignant tumor, advanced liver disease, renal failure, autoimmune diseases, and other inflammatory diseases and women who were pregnant or breast-feeding were excluded from the study. Patients in the UA group with stable angina and patients in the AIS group with acute cerebral hemorrhage, subarachnoid hemorrhage, and stable or unstable angina pectoris were excluded from the study. Control subjects (*n* = 20) were healthy volunteers recruited from the same population and the same area of China as the patients’ group. Five subjects in every group were randomly allocated in microarray profiling and the other 15 subjects underwent qRT-PCR verification.

### Plasma collection and RNA isolation

Whole blood samples (10 mL) were drawn from each of the 60 participants (20 from the UA group, 20 of AIS group and 20 healthy volunteers/blank group) with a 19-gauge needle for the clean venipuncture of antecubital vein on the morning following hospitalization. The blood samples were collected within 24 h of the onset of chest pain in UA patients and within 24 h of the hospitalization in AIS patients. Blood was drawn into EDTA-containing tubes and peripheral blood mononuclear cells (PBMCs) were isolated by density gradient centrifugation with Ficoll (Invitrogen, Carlsbad, CA, USA). Total RNAs were extracted from PBMCs using Trizol reagent (Invitrogen) according to the manufacturer’s instructions. RNA quantity and purity were assessed using NanoDrop ND-1000 (Thermo Scientific, Waltham, MA, USA). Passing criteria for absorbance ratios were established at A260/A280≥1.8 and A260/A230≥1.5 indicating acceptable RNA purity. RNA Integrity Number (RIN) values were ascertained using Agilent RNA 6000 Nano assay (Agilent Technologies, Santa Clara, CA, USA). Passing criteria for the RIN value were established at ≥6 indicating acceptable RNA integrity. Genomic DNA contamination was evaluated by gel electrophoresis. The RNA samples were stored at −80 °C before analysis.

### mRNA expression profiling

Total RNAs were used for mRNA expression profiling by the Human Whole Genome OneArray v5 (Phalanx Biotech Group, Hsinchu, Taiwan). It contained 30,275 DNA oligonucleotide probes, and each probe was a 60-mer designed in the sense direction. Approximately 29,187 probes corresponded to the annotated genes in RefSeq v38 and Ensembl v56 database, and 1088 probes were control probes. Fluorescent aRNA targets were prepared from 1 or 2.5 μg total RNA samples using the OneArray Amino Allyl aRNA Amplification Kit (Phalanx Biotech Group) and Cy5 dyes (Amersham Pharmacia, Piscataway, NJ, USA). Fluorescent targets were hybridized to the Human Whole Genome OneArray with Phalanx hybridization buffer using the Phalanx Hybridization System. After 16 h of hybridization at 50 °C, nonspecific binding targets were washed away by three different washing steps (Wash I 42 °C 5 min; Wash II 42 °C 5 min, 25 °C 5 min; Wash III rinse 20 times), and the slides were dried by centrifugation and scanned by an Axon 4000B scanner (Molecular Devices, Sunnyvale, CA, USA). The intensities of each probe were obtained by GenePix 4.1 software (Molecular Devices). The raw intensity of each spot was loaded into Rosetta Resolver System (Rosetta Biosoftware, Seattle, WA, USA) to process data analysis. The error model of Rosetta Resolver System could remove both systematic and random errors form the data. Those probes with background signals were filtered out. Probes that passed the criteria were normalized by a 50 % median scaling normalization method. Normalized spot intensities were transformed to mRNA expression log2 ratios.

### microRNA expression profiling

miRNA expression profiling was performed in the same set of samples in the mRNA microarray analysis. The Human miRNA OneArray v4 (Phalanx Biotech Group) was used. It contained triplicated 1884 unique miRNA probes from humans (miRBase Release v18), each printed in technical triplicate, and 144 experimental control probes. Small RNA was pre-enriched by Nanoseplook (Pall Corporation, Port Washington, NY, USA) from 2.5 μg total RNA samples and labeled with the miRNA ULS Labeling Kit (Kreatech Diagnostics, Vierweg, Amsterdam, The Netherlands). Labeled miRNA targets were hybridized to the Human miRNA OneArray v4 with OneArray Hybridization System. After 16 h of hybridization at 37 °C, nonspecific binding targets were washed away by three different washing steps (Wash I 37 °C 5 min; Wash II 37 °C 5 min, 25 °C 5 min; Wash III rinse 20 times), and the slides were dried by centrifugation and scanned by an Axon 4000B scanner (Molecular Devices). The Cy5 fluorescence intensities of each probe were analyzed by GenePix 4.1 software (Molecular Devices). The raw intensity of each probe was processed by the R program. Probes that passed the criteria were normalized by the 75 % median scaling normalization method. Normalized spot intensities were transformed to miRNA expression log2 ratios.

### Identification of differentially expressed genes

The Limma package [26] in R software (Version 3.1.1) was used to compare the normalized probeset intensities between the blank group and the UA group as well as the blank group and the AIS group. The threshold of miRNA comparison was set as *P* < 0.05 and fold change >0.8, while the threshold of mRNAs comparison was set as *P* < 0.05 and fold change >1 (to concentrate on those mRNAs that interact more with miRNAs). The Annotate package was used to annotate the differentially expressed genes (DE genes).

### Bioinformatic analysis

To identify the pivotal and characteristic genes of BSS, the DE mRNAs and miRNAs of the blank group vs. the UA group and the blank group vs. the AIS group were pooled together for profound analysis. The overlapped mRNAs and miRNAs of two sets of DE genes were identified. Database for Annotation, Visualization, and Integrated Discovery (DAVID) [27] online tools (http://david.abcc.ncifcrf.gov/home.jsp) was applied to annotate and analyze the GO enrichment of DE genes. The threshold was set as *P* < 0.05. MiRWalk 2.0 database [28] was used to predict the target mRNAs of overlapped miRNAs. The DIANA-mT, miRanda, miRWalk and TargetScan programs were applied. If the prediction was identified in no less than 3 programs, the prediction was considered solid. Those targeted miRNA-mRNAs found in both overlapped DE mRNA and miRNA sets were screened out. STRING (Version 9.1) online tools (http://www.string-db.org/) and Cytoscape (Version 3.1.1) [29] were used to construct and visualize the mRNA-GO terms network and the miRNA-mRNA targeting network.

### qRT-PCR validation of pivotal mRNAs and miRNAs

With the bioinformatics analysis of the DE mRNAs and miRNAs, miR-146b-5p and -199a-5p were the pivotal genes in the BSS-associated characteristic network and merited further investigation. Thus, real-time quantitative polymerase chain reaction (qRT-PCR) of miR-146b-5p and miR-199a-5p was performed to validate the bioinformatics analysis results in an independent cohort of 15 UA patients, 15 AIS patients and 15 healthy controls in accordance with the microarray subjects.

QRT-PCR was performed according to the manufacturer’s instructions (System Biosciences, Mountain View, CA, USA) with 2X SYBR Green qPCR Mastermix (Roche Applied Science, Indianapolis, IN, USA) and a 7900HT Fast Real-Time PCR System (Applied Biosystems, Carlsbad, CA, USA). The primer sequences of miR-146b-5p, miR-199a-5p and Human U6 snRNA (internal control) were as follows: miR-146b-5p: forward 5′-TGA GAA CTG AAT TCC ATA GGC T-3′; miR-199a-5p: forward 5′-CCC AGT GTT CAG ACT ACC TGT TC-3′; Human U6 snRNA: forward 5′-CGC AAG GAT GAC ACG CAA ATT C-3′. The thermal cycling conditions were 95 °C for 5 min, followed by 40 cycles of 95 °C for 30 s, 55 °C for 30 s, 72 °C for 50 s, and a final extension at 72 °C for 8 min. The relative expression fold change of target genes was calculated using the comparative CT method with the 2^−ΔΔCT^ equation.

## Results

### Clinical characteristics of participants

A total of 60 subjects were recruited for this study and divided into 2 cohorts. The microarray cohort included 5 UA patients, 5 AIS patients and 5 healthy controls. The qRT-PCR validation cohort included 15 UA patients, 15 AIS patients and 15 healthy controls. The clinical characteristics of the 2 cohorts were listed in Tables 2 and 3. The 3 groups in both cohorts were matched in terms of age, sex, BMI (body mass index), CRP (C-reactive protein) and most of the blood lipids (*P* > 0.05).

**Table 2:** Clinical characteristics of microarray subjects

	Controls (*n* = 5)	UA (*n* = 5)	AIS (*n* = 5)	*P* value
Male (n)	3	3	3	
Age (years)	52.60 ± 7.40	56.40 ± 6.95	58.00 ± 7.18	0.571
BMI (kg/m2)	23.24 ± 1.62	23.18 ± 3.89	22.58 ± 2.76	0.320
BSS Grades	0	72.80 ± 10.47	78.60 ± 2.61	0.000
Hypertension (n)	0	4	5	0.000
Type 2 diabetes mellitus (n)	0	0	0	
Total cholesterol (mmol/L)	4.02 ± 0.79	4.18 ± 0.89	4.24 ± 0.77	0.964
LDL cholesterol (mmol/L)	2.51 ± 0.60	2.33 ± 1.01	2.74 ± 0.63	0.871
HDL cholesterol (mmol/L)	1.29 ± 0.30	1.33 ± 0.35	1.04 ± 0.17	0192
Triglycerides (mmol/L)	1.09 ± 0.34	1.15 ± 0.50	1.79 ± 0.88	0.247
CRP (mg/L)	1.82 ± 0.78	2.84 ± 1.74	3.37 ± 1.66	0.316

**Table 3:** Clinical characteristics of qRT-PCR subjects

	Controls (*n* = 15)	UA (*n* = 15)	AIS (*n* = 15)	*P* value
Male (*n*)	10	11	9	0.838
Age (years)	56.87 ± 6.70	57.53 ± 6.33	53.07 ± 5.87	0.182
BMI (kg/m2)	21.80 ± 2.20	23.85 ± 1.81	22.53 ± 2.78	0.092
BSS Grades	0	68.60 ± 8.72	67.93 ± 9.88	0.000
Hypertension (*n*)	0	11	14	0.000
Type 2 diabetes mellitus (*n*)	0	6	3	0.018
Total cholesterol (mmol/L)	3.71 ± 0.76	4.28 ± 0.89	4.60 ± 0.87	0.072
LDL cholesterol (mmol/L)	2.37 ± 0.54	2.66 ± 0.63	2.92 ± 0.64	0.056
HDL cholesterol (mmol/L)	1.37 ± 0.29	1.31 ± 0.31	1.14 ± 0.19	0.112
Triglycerides (mmol/L)	1.03 ± 0.28	2.46 ± 1.03	1.82 ± 1.09	0.000
CRP (mg/L)	3.97 ± 1.86	4.84 ± 2.10	4.57 ± 2.34	0.682

### DE mRNAs and miRNA identification

The DE mRNAs and miRNAs between the blank group and the UA group and the blank group and the AIS group were screened out via the limma package in R software. In the comparison between the blank group and UA group, there were 1151 DE mRNAs and 27 DE miRNAs. Among these, 731 (63.51 % of all the DE mRNAs) mRNAs were up-regulated in the UA group and 420 (36.49 % of all DE mRNAs) mRNAs were down-regulated. Meanwhile, 25 (92.6 % of all the DE miRNAs) miRNAs were up-regulated and 2 (7.4 % of all the DE miRNAs) miRNAs were down-regulated. In the comparison between the blank group and AIS group, there were 585 DE mRNAs and 22 DE miRNAs. Among these, 416 (71.11 % of all the DE mRNAs) mRNAs were up-regulated in the AIS group and 169 (28.89 % of all DE mRNAs) mRNAs were down-regulated. All of the DE miRNAs were up-regulated and none of the miRNAs were down-regulated.

### Overlapped DE miRNAs and mRNA identification

The two sets of DE mRNAs and miRNAs represent the characteristics of BSS in two different diseases. Apart from the differences of UA and AIS, there were similarities between those two groups of patients. Both of them were diagnosed with BSS in the perspective of TCM. To seek the intersections of UA and AIS is to seek the general and common features of BSS. In the theory of TCM, it is called “the same syndrome in different diseases.” To this end, we pooled the two sets of DE mRNAs and miRNAs together and screened out the overlapped mRNAs and miRNAs. There were 304 up-regulated mRNAs, 97 down-regulated mRNAs and 11 up-regulated miRNAs, from which may we find the common features and pathological mechanisms of BSS and provide potential biomarkers in the diagnosis of BSS.

### Functional analysis of overlapped DE miRNAs and mRNAs

**Fig. 1:**
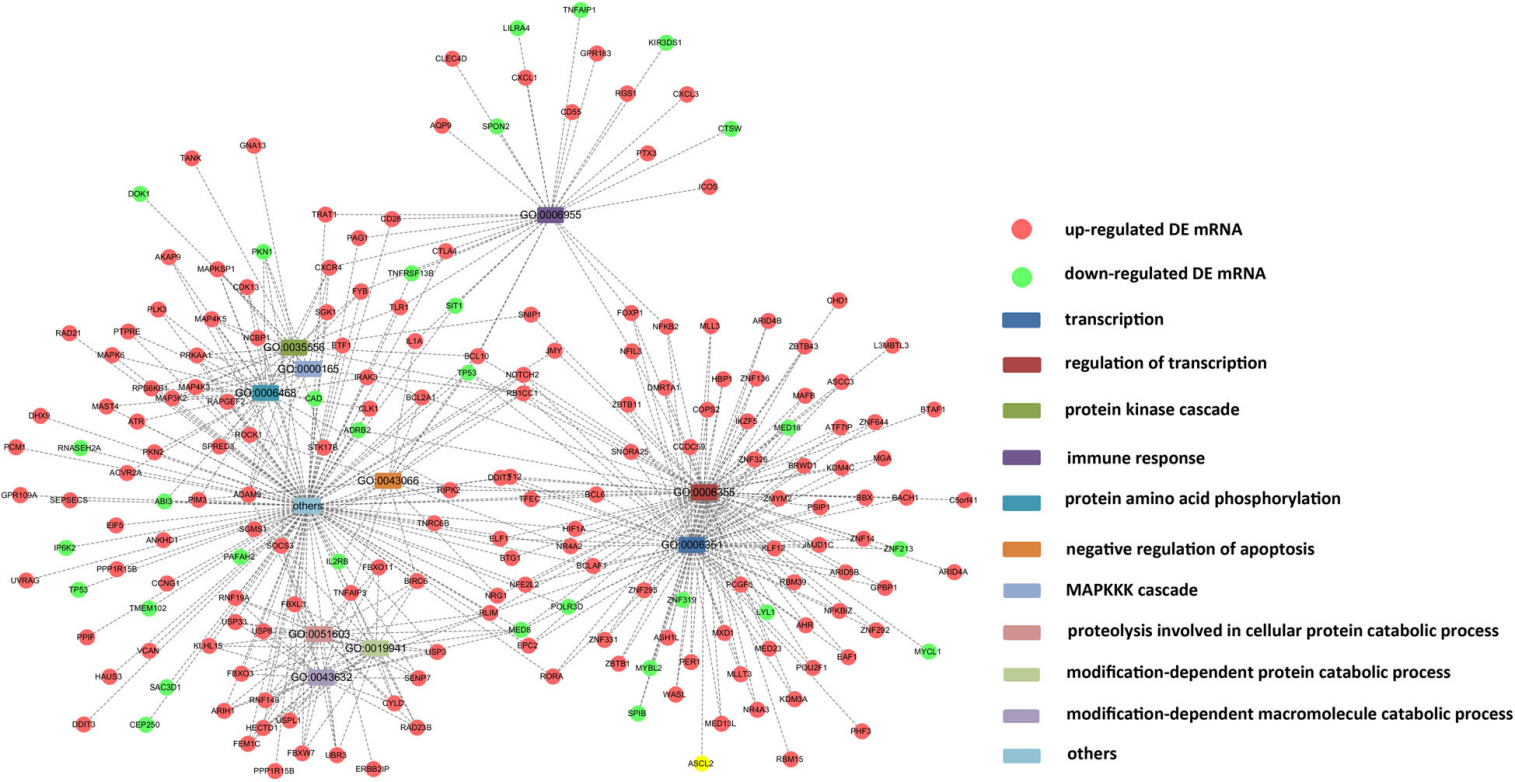
The functional network of GO terms and the involved DE mRNAs

The total of 401 overlapped DE mRNAs were uploaded to DAVID online tools to analyze the enrichment of GO terms. A total of 31 GO terms were significantly enriched. The details of the GO terms were listed in Table 4. The functional network of GO terms and the involved DE mRNAs was constructed and visualized via cytoscape (Fig. 1).

**Table 4:** Details of the significantly enriched GO terms

Term	*P*-Value
transcription	0.000087
regulation of transcription	0.00011
protein kinase cascade	0.00038
immune response	0.0027
protein amino acid phosphorylation	0.0032
negative regulation of apoptosis	0.0036
MAPKKK cascade	0.01
proteolysis involved in cellular protein catabolic process	0.012
modification-dependent protein catabolic process	0.014
modification-dependent macromolecule catabolic process	0.014
phosphorylation	0.018
regulation of apoptosis	0.019
regulation of T cell receptor signaling pathway	0.021
ER overload response	0.021
regulation of programmed cell death	0.022
regulation of cytokine production	0.025
cellular macromolecule catabolic process	0.029
regulation of antigen receptor-mediated signaling pathway	0.03
centrosome organization	0.037
regulation of leukocyte activation	0.039
cellular response to stress	0.039
regulation of translation	0.039
toll-like receptor signaling pathway	0.04
negative regulation of immune system process	0.043
response to endoplasmic reticulum stress	0.043
positive regulation of skeletal muscle growth	0.046
microtubule organizing center organization	0.046
ER-nuclear signaling pathway	0.046
JNK cascade	0.047
anti-apoptosis	0.05
regulation of cell size	0.05

### Target prediction of overlapped DE miRNAs and mRNAs

**Fig. 2:**
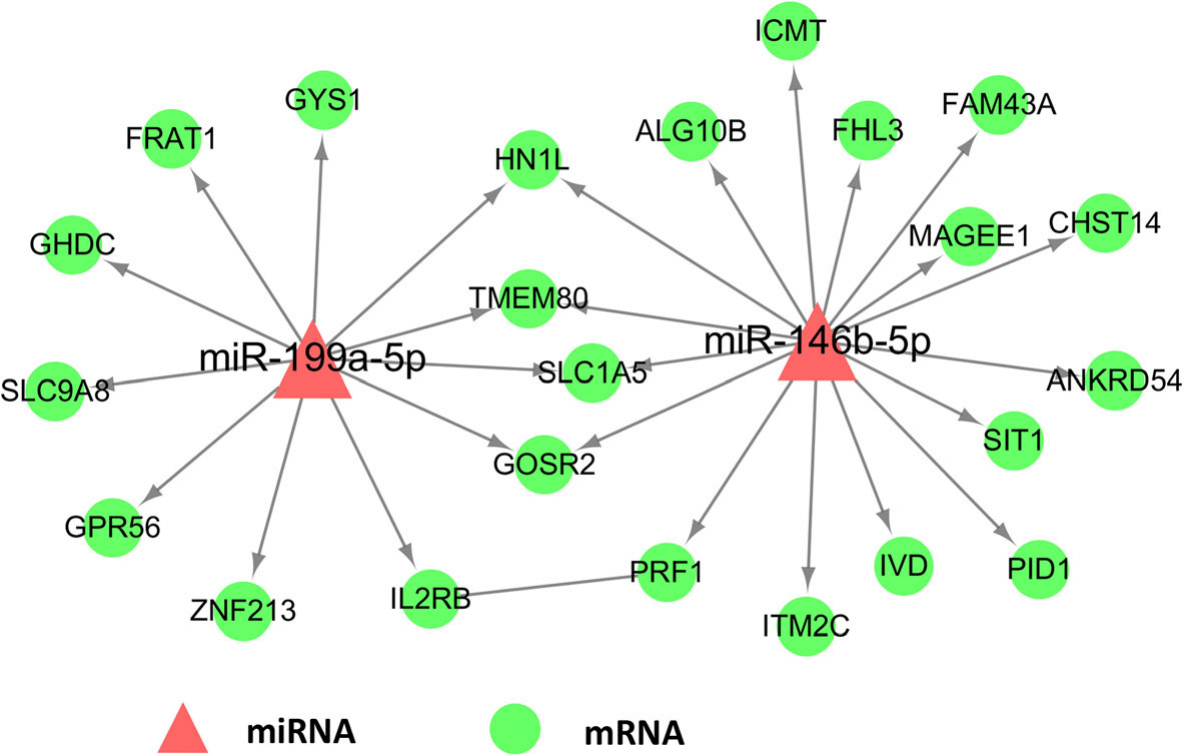
The miRNA-mRNA interactions

MiRWalk was used to predict the target mRNAs of the 11 up-regulated miRNAs, and thousands of miRNA-mRNA targeting predictions were obtained. miRNAs were usually negatively correlated with target mRNAs. Since all the overlapped DE miRNAs were up-regulated, we cross-referenced the predicted mRNA with the down-regulated DE mRNA. Eventually, we identified 2 miRNAs and 23 targeted DE mRNAs as the miRNA-mRNA targeting network of BSS. The visualization of miRNA-mRNA interactions is shown in Fig. 2.

### qRT-PCR validation of DE miRNAs

**Fig. 3:**
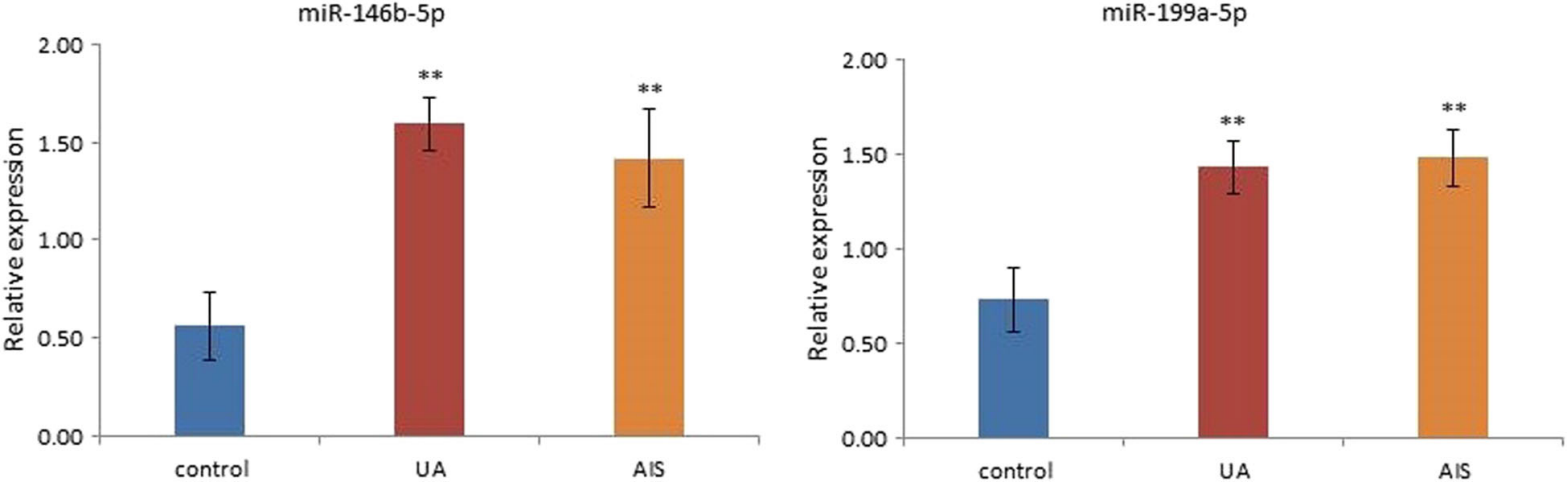
The qRT-PCR validation of miR-146b-5p and miR-199a-5p

The qRT-PCR results of miR-146b-5p and miR-199a-5p in the validation cohort are presented in Fig. 3. miR-146b-5p and miR-199a-5p were upregulated both in the UA and AIS groups compared to the healthy control group (*P* < 0.01). Such results coincided with the microarray profiling and bioinformatics analysis.

## Discussion

TCM has been proven to be effective as complementary and alternative medicine in treating cerebrovascular and cardiovascular diseases. The key to utilizing TCM appropriately and maximizing its efficacy is to accurately apprehend syndrome differentiation. Therefore, a standard diagnostic criterion with objective indexes is needed. Comprehensive whole-genome screening of the transcriptome has previously suggested that disease-specific mechanisms are distinguishable [30–32]. Syndrome differentiations could be characterized by the expression of different genes [17–22]. Using microarray profiling and bioinformatics to investigate the gene expression patterns of BSS could illuminate the pathological mechanisms of BSS and provide potential biomarkers for clinical diagnosis.

Recent studies have demonstrated that miRNAs can circulate in systemic blood in a remarkably stable form [33]. It is possible to use the circulating miRNA expression patterns as biomarkers for the differentiation of syndromes such as BSS. With the auxiliary miRNA’s target genes, a syndrome-related miRNA-mRNA network could be constructed.

In this study, the microarray profiling comparison between the blank group and the UA group showed pathological changes of UA from the genomic level, along with the blank group and AIS group. The DE mRNAs and miRNAs indicated the characteristics of UA and AIS with BSS. When we focused on the similarities of BSS in different diseases (the same syndrome in different diseases), we merged and screened out the overlapped DE mRNAs and miRNAs in the two comparisons. The 401 overlapped DE mRNAs and 11 miRNAs represent the genomic characteristics of BSS beyond diseases. The GO analysis of the 401 overlapped DE mRNAs showed the biological processes involved in BSS. Basic life activities and processes were the major enriched terms, including *transcription* (GO:0006351, *P* = 0.000087), *regulation of transcription* (GO:0006355, *P* = 0.00011), *protein kinase cascade* (GO:0035556, *P* = 0.00038), and *protein amino acid phosphorylation* (GO:0006468, 0.0032). Among the top 10 GO terms sorted by *P*-value, *immune response* (GO:0006955, *P* = 0.0027) and *negative regulation of apoptosis* (GO:0043066, *P* = 0.0036) might be the pivotal pathological processes related to BSS.

The 11 overlapped DE miRNAs were the candidates for biomarkers of BSS. Among the multiple functions and targets of miRNA, cardiovascular- and cerebrovascular-related studies were searched specifically. *a) miR-221-3P*: miR-221-3p was up-regulated in the patients with ischemic stroke [34] and acute coronary syndrome and was correlated with plasma and platelets [35]. *b) miR-150*: miR-150 was significantly related to immune response, and its increase in serum was correlated with the elevation of antibody titers [36]. A previous study reported that miR-150-5p, miR-1275, and miR-365a-3p were associated with chronic heart diseases and reflected the dysregulation of B-cell-centered immune function [37]. It was reported that variations in the expression of miR-146a, miR-150, and miR-155 in MI compared to normal hearts were detected, which are involved in the regulation of innate immunity [38]. *c) miR-146a-5p*: miR-146a-5p has been identified as a negative regulator in the innate immune and inflammatory responses mediated by Toll-like receptor (TLR) 4 and polymorphisms (miRSNPs) [39, 40]. Research on miR-146b-5p has shown that it could reduce the expression of TNFα via the IRAK/NFkB pathway [39, 41]. It was reported that miR-146b-5p is linked to the regulation of both apoptotic and anti-apoptotic genes [42]. *d) miR-199a-5p*: Roncarati R et al*.* compared hypertrophic cardiomyopathy (HCM) patients with healthy controls, and the peripheral plasma levels of miR-199a-5a and other miRNAs were detected. miR-199a-5p was significantly increased and correlated with hypertrophy [43]. These studies helped confirm the possible relationship between the 11 miRNAs and AIS and IHD and indicated the involved functional processes.

To increase the credibility of miRNA as the biomarker of BSS, the combination of miRNA and targeted mRNA was applied. Among the 11 overlapped DE miRNAs, only miR-146b-5p and miR-199a-5p had targeted mRNAs, which were also present in the overlapped DE mRNAs. Additionally, qRT-PCR validated the upregulation of miR-146b-5p and miR-199a-5p in BSS patients. Previous studies on miR-146b-5p and miR-199a-5p demonstrated the possible mechanisms of BSS in cerebrovascular and cardiovascular diseases. In this study, miR-146b-5p, miR-199a-5p and their 23 targeted overlapped DE mRNAs formed a biomarker network for the diagnosis of BSS. The pivotal roles of miR-146b-5p and miR-199a-5p made them the center of the diagnostic network of BSS, and the mRNAs validated the diagnosis.

The present study revealed that circulating miRNAs and mRNAs might be the potential biomarkers of BSS, and the comprehensive application of miRNAs and mRNAs as a network might enhance the credibility of miRNAs and mRNAs as BSS diagnostic criteria. MiR-146b-5p and miR-199a-5p were the key miRNAs in the diagnostic network, which might be the most valuable biomarkers for BSS. With the help of these objective indexes, the diagnostic criteria of BSS could be more advanced and the apprehension of BSS diagnosis may be easier for practitioners.

Apart from the potential clinical applications this study has provided, it presented some limitations. The sample sizes of both cohorts in this study were small, especially for the microarray profiling cohort. The transcriptomic analysis from such small sample sizes may lead to fragmentary results and affect the following validation cohort. The identified DE genes for BSS are preliminary, the application in clinical practice was not evaluated fully in this study. Further improvement and retrenchment on the diagnostic network are required. Clinical studies with more BSS participants are required to extensively evaluate the miRNAs and mRNAs as feasible biomarkers. Nevertheless, this study laid the groundwork for identifying miR-146b-5p, miR-199a-5p, and those targeted mRNAs as potential network-type biomarkers for BSS. The diagnosis of BSS could be achieved more objectively if further research could validate these results in a larger cohort of participants.

## Conclusions

In general, this study presented a new approach to identify potential biomarkers for BSS via genomics and bioinformatics. miR-146b-5p, -199a-5p and the 23 targeted mRNAs formed a diagnostic network for BSS. With further improvement and the validation of this diagnostic network, more objective diagnostic criteria for BSS might be utilized in clinical practice.

## Abbreviations

ACC/AHA: American College of Cardiology/American Heart Association
AHA/ASA: American heart association/American stroke association
AIS: Acute ischemic stroke
AS: Atherosclerosis
BMI: Body mass index
BSS: Blood-stasis syndrome
CAD: Coronary artery disease
DAVID: Database for Annotation, Visualization, and Integrated Discovery
DE genes: Differential expressed genes
GO: Gene ontology
IHD: Ischemic heart disease
IS: Ischemic stroke
miRNA: microRNA
mRNA: Messenger RNA
TCM: Traditional Chinese medicine
UA: Unstable angina
WHO: World Health Organization
