# Erratum to: Identification of more objective biomarkers for Blood-Stasis syndrome diagnosis

**DOI:** 10.1186/s12906-016-1448-7

**Published:** 2016-11-10

**Authors:** Jiangquan Liao, Yongmei Liu, Jie Wang

**Affiliations:** 1 Graduate School, Beijing University of Chinese Medicine, No.11 Beisanhuan East Road, Chaoyang District, Beijing 100029 China; 2 Guang’anmen Hospital, China Academy of Chinese Medical Sciences, No.5 Beixiange Street, Xicheng District, Beijing 100053 China

## Erratum

Following publication of the original article [1] it was brought to our attention that the sequence of the author affiliations was incorrectly ordered. In the original article the sequence appears as follows:

^1^Guang’anmen Hospital, China Academy of Chinese Medical Sciences, No.5 Beixiange Street, Xicheng District, Beijing 100053, China

^2^Graduate School, Beijing University of Chinese Medicine, No.11 Beisanhuan East Road, Chaoyang District, Beijing 100029, China

However, the corrected sequence should appear as:

^1^Graduate School, Beijing University of Chinese Medicine, No.11 Beisanhuan East Road, Chaoyang District, Beijing, 100029 China

^2^Guang’anmen Hospital, China Academy of Chinese Medical Sciences, No.5 Beixiange Street, Xicheng District, Beijing, 100053 China

Please also note that based on this new sequence, the author affiliations should also be updated to the following:

Jiangquan Liao^1,2^, Yongmei Liu^2^ and Jie Wang^2*^
